# Effects of long-term (70 years) nitrogen fertilization and liming on carbon storage in water-stable aggregates of a semi-arid grassland soil

**DOI:** 10.1016/j.heliyon.2021.e08690

**Published:** 2021-12-28

**Authors:** Kwenama Buthelezi, Nkosinomusa Buthelezi-Dube

**Affiliations:** a School of Agricultural, Earth and Environmental Sciences, University of KwaZulu-Natal, P. Bag X01, Scottsville, Pietermaritzburg, 3201, South Africa; b Soil Science, School of Agricultural, Earth and Environmental Sciences, University of KwaZulu- Natal, Private Bag X01, Scottsville, South Africa

**Keywords:** Carbon storage, Mean weight diameter, Lime, Ammonium sulphate, Ammonium nitrate, Soil aggregates

## Abstract

Grasslands cover up to 40.5% of the world's landmass and store 30% terrestrial carbon (C). Various practices, including mineral fertilization and liming, are used to manage these ecosystems with potential long-term effects on the size and distribution of soil aggregates and inevitably carbon dynamics. The objective of this study was to examine the long-term effects of ^nitrogen fertilization and liming on soil carbon storage and its dynamics in water-stable^ aggregates of a semi-arid grassland. Soil samples (0–10 cm) were collected from Ukulinga long-term grassland trial in Pietermaritzburg, South Africa where nitrogen fertilizers have been applied annually and lime every five years for 70 years. Ten treatments were studied: the control (0 kgN/ha and unlimited), lime at 2250 kg/ha (L), ammonium sulphate at 70 kg/ha (AS70) and 211 kg/ha (AS211); ammonium nitrate at 70 kg/ha (AN70) and 211 kg/ha (AN211); AS70 + lime (AS70L); AS211 + lime (AS211L); AN70 + lime (AN70L) and AN211 + lime (AN211L).

Nitrogen fertilizers significantly reduced soil pH and increased total soil N. Liming increased soil pH with no effect on total soil N. Lime and lime + N fertilizer treatments had no effect on mean weight diameter (MWD) while separate N application decreased MWD and large macro-aggregates (LMA). Lime only treatment had no effect on water stable aggregate (WSA) fractions. Nitrogen fertilization and liming (separately or in combination) did not affect total C concentration and stocks. Overall, soils had very high total soil organic carbon ranging from 49.7 – 57.6 g/kg across treatments. Nitrogen fertilization decreased organic carbon in LMA in AS70 (1.52%) and AN211 (1.67%) treatments compared to the control (3.40%) which was in concert with increases in C associated with small macro-aggregates (SMA) and micro-aggregates (MiA and SCA). Organic carbon in SMA was 2.67 % (AS70); AS211 (2.62 %); AN70 (2.02 %); AN211 (2.49 %) compared to 1.26 % in the control. Lime + N fertilizer treatments increased C storage in all aggregate fractions compared to N fertilizer only treatments. The lack of response in total SOC to 70 years of N fertilization and liming suggests possible C saturation given the high soil C concentration. Changes in C associated with WSA fractions suggests their importance as diagnostic indicators of N fertilization and liming induced changes in SOC. Findings also show that ammonium-based N fertilization is associated with soil acidification, dispersion of LMA resulting in an increase of microaggregates and C stored in them. Liming can counteracts acidifying and the dispersive effect on NH_4_^+^ associated with ammonium-based fertilizers thus restoring macro-aggregation in N fertilized grasslands. These findings suggests that long-term N addition may result in poor soil physical condition and possible stabilization of C in stable fractions.

## Highlights

• Long-term N addition and liming did not affect bulk SOC content and stocks but lead to redistribution of C into water stable aggregates.
• Long-term N fertilization caused soil acidification and reduced mean weight diameter.
• Long-term N fertilization increased micro-aggregates and SOC stored in them.
• Long-term liming did not affect WSA, and C stored in them.
• Liming improved aggregation and increased C associated with large macro aggregates when co-applied with N fertilizers.

## 1 Introduction

Grasslands are considered among the worlds’ largest ecosystems consisting of rangelands, shrublands, pastureland and croplands sown with pasture and fodder crops (Rumpel et al., 2015). In total, these ecosystems are estimated to cover up to 52.5 million square kilometres (Suttie et al., 2005), which make up to 40.5 % of the terrestrial area. Grasslands store about 30 % of terrestrial C (Cenini et al., 2015) and contribute significantly to carbon and nutrient cycling. Management practices such as liming and nitrogen fertilization, widely used on often N-limited grasslands, affect soil physicochemical properties of this ecosystem. Nitrogen (N) fertilization increases N availability and plant growth thus increasing above-ground C storage. Nitrogen availability has a major control on soil C cycling and storage (Averill and Waring, 2018).

Inconsistent results have been reported with regards to total SOC in response to N addition with some studies reporting no response (Hassink, 1994; Zeglin et al., 2007; Lu et al., 2011), decrease (Luo et al., 2019) and increase (Du et al., 2014; Cenini et al., 2015; Eze et al., 2018; Crème et al., 2020; Glendining et al., 1996). No response of total SOC has been attributed to decoupling of above-ground and below-ground C dynamics due to complex C related processes associated with N addition causing lack of soil C storage (Lu et al., 2021). High above-ground C storage and organic inputs to soils does not always result in increased soil C storage suggesting a divergent below-ground C process (Lu et al., 2011). In addition, some soils have been found to be carbon saturated with respect to C inputs (Stewart et al., 2007). High C inputs and/or high soil C levels have been associated with decreased bulk soil C stabilization in long-term agroecosystem experiments (Nyborg et al., 1995; Reicosky et al., 2002; Gulde et al., 2008) suggesting a saturation level for soil C (Six et al., 2002). Nitrogen availability together with high plant tissue N due to N addition accelerates soil organic matter decomposition explaining a decrease in soil C observed in some studies. On the other hand, N addition can slow microbial activity and litter decomposition resulting in enhanced C input and reduced C output resulting in increased C storage (Buchkowski et al., 2015; Chen et al., 2020). Size and turnover of labile and recalcitrant C pools are largely influenced by differences in soil management, ecosystems and soil properties making it difficult to predict the net effect of N enrichment on soil C storage (Neff et al., 2002). According to Averill and Waring (2018) interaction among plants, microbes and soil properties as well as control by microbial physiology, soil mineralogy and acidity can explain observed contrasting effects of N addition on soil C dynamics. Moreover, Schmidt et al. (2011) showed the persistence of organic carbon in ecosystems as being a function of complex interactions between organic matter and its environment.

Grasslands and pastures are subjected to acidic conditions due to fertilizer application or extensive cation leaching (Paradelo et al., 2015). Application of ammonium-based fertilizers are particularly responsible for significant soil acidification in human-managed pastured. The N-induced acidity results in loss of basic cations and high aluminum toxicity. Loss of Ca due to N-induced acidity interferes with the bridging of clays and organic materials thus decreasing stability of water stable aggregates (Oades, 1984). Exchangeable Ca contributes significantly to aggregation through flocculation of clay particles (Rengasamy and Marchuk, 2011). Moreover, fertilization-induced release of NH_4_^+^ is associated with reduced aggregate stability (Haynes and Naidu, 1998). Adsorption of NH_4_^+^ (low hydration energy) in place of cations with high hydration energy (e.g. Mg^2+^ and Ca^2+^) results in the collapse of the interlayer (Rigol et al., 1999) due to NH_4_^+^-induced dispersion (Blanco-Canqui, 2020). Liming is employed to remediate the effect of acidity on soil's physical and chemical properties. A review by Paradelo et al. (2015) showed that several factors determine the net effect of liming on SOC. For example, (a) liming can increase microbial activity by creating more favourable pH conditions, which will enhance OM mineralization, thus decreasing SOC stocks (b) favourable pH condition increases plant productivity and net primary production (NPP) resulting in more significant OM inputs in the form of dead roots and decaying crop residues consequently increasing SOC and (c) liming is known to ameliorate soil structure thus increasing clay assemblages and clay-organic matter bonds creating physical and physico-chemical protection of SOC.

Stabilization of organic matter in soil aggregates is the principal mechanism for long-term soil C sequestration (Verchot et al., 2011; Mosquera-Losada et al., 2015). Soil aggregation is one of the main factors that encourage the persistence of SOC by forming a protecting barrier that impedes microbial access and increases water filtration, thus reducing water runoff and C losses through erosion (Bai et al., 2021). Generally, increases in SOM are associated with C-rich macro-aggregates but long-term C sequestration depends on stabilization of carbon in micro-aggregates (Tisdall and OADES, 1982; Six et al., 2000a, Six et al., 2000b). According to Wu et al. (2004) soil organic carbon (SOC) in different size aggregates characterizes the relationship between organic matter balance and mineralization rate with dual significance in soil fertility and carbon sink. Organic C stored in differently sized stable aggregates has shown a strong response to management compared to bulk SOC (Denef et al., 2007; Wang et al., 2015). For example, Chen et al. (2020) reported a significant increase in mineral associated C while no response was observed for bulk SOC following N addition to B. platyphylla *forest*. According to Lu et al. (2021) the combined effects of individual aggregate C drives the general response of soil C pools to N enrichment. This suggests that separating bulk soil into different functional C pools may be used as a diagnostic indicator for potential changes and reveal observed complex responses of SOC to N addition. Investigating carbon pools in soil aggregates thus provides an understanding of the dynamics of carbon sequestration and mineralization in aggregates (Whalen and Chang, 2002).

Despite many studies on the effect of N enrichment on bulk soil C dynamics, there is lack of evidence on the mechanisms explaining the response of soil aggregates (Lu et al., 2021). In addition, most long-term studies on grassland management focus on separate effects of liming and nitrogen on SOC, C in aggregates and aggregates stability while little attention has been given to their interaction effects on these parameters. For example, Johnson et al. (2005) has shown that a combination of lime and N affect soil microbial biomass, soil pH, and total N in a short-term upland grassland experiment in Scotland but with inconsistent results due to differences in climate, soil properties, and type of fertilizer. Moreover, similar studies from Ukulinga veld fertilization experiment in South Africa, focused on plant productivity (Le Roux and Mentis, 1986), plant diversity (Grunow et al., 1970; Scott and Rabie, 1956), soil respiration (Ward et al., 2017b), and soil biology (Zeglin et al., 2007) as affected by fertilizer and lime addition. Soil properties such as SOC, MWD, and aggregate stability are reported as essential soil characteristics and not profoundly examined on the possible processes involved. This paper aims to examine the long-term effects of nitrogen fertilization and liming on water-stable aggregates (WSA) fractions and carbon stored in them. We hypothesize that; (1) Nitrogen application will increase carbon concentration in bulk soil and decrease WSA because of N-induced effects on soil pH and aggregation; (2) Liming will increase the WSA, MWD, and increase C concentration in macro-aggregates.

## 2 Material and methods

### 2.1 Study site

**Figure 1:**
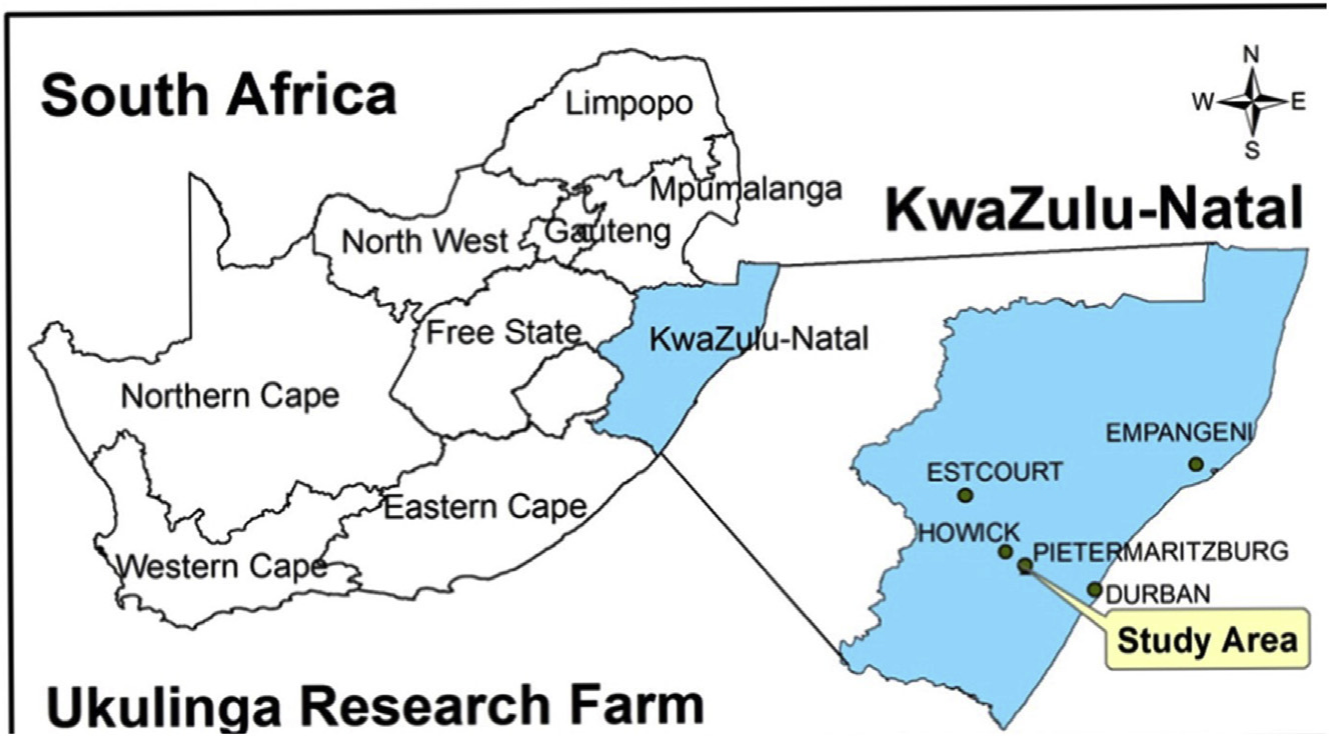
Location of the long-term grassland study site in South Africa. (Abdalla et al., 2016).

The study was conducted in a long-term grassland trial located at the University of KwaZulu Natal Ukulinga research farm, in Pietermaritzburg, South Africa (29° 24′E, 30° 24′S) (Figure 1). The area is semi-arid with mean annual precipitation of 790 mm and located on a plateau at 838 m a.s.l. (Ward et al., 2017b, 2020). The vegetation of the area is southern tall grass veld or, at a larger spatial scale, KwaZulu-Natal hinterland thornveld, which is an open savanna of Acacia Vachellia sieberiana with patches of Hyparrhenia hirta L. and other herbaceous species (Ward et al., 2017d). The soil is a Westleigh form, derived from localized dolerite intrusions into a shale parent material (Rutherford et al., 2006).

### 2.2 Experimental design

The experiment was established in 1951 with the application of nitrogen, phosphorus, and lime as the main treatments and had the average pH of 5.7 (Scott and Rabie., 1956). This study focused on nitrogen, applied annually as ammonium nitrate and ammonium sulphate at 70 and 211 kg N/ha and dolomite lime applied at 2250 kg/ha every five years. The experiment was laid out in a randomized block design with 9.0 × 2.7 m size plots replicated three times (Morris and Fynn, 2001; Ward et al., 2017d). The following treatments were selected: **1)** Control (0 kg lime or N fertilizer/ha); **2)** lime at 2250 kg/ha (L); **3)** ammonium sulphate at 70 kg/ha (AS70); **4)** ammonium sulphate at 211 kg/ha (AS211); **5)** ammonium nitrate at 70 kg/ha (AN70); **6)** ammonium nitrate at 211 kg/ha (AN211); **7)** ammonium sulphate at 70 kg/ha + lime (AS70L); **8)** ammonium sulphate at 211 kg/ha + lime (AS211L); **9)** ammonium nitrate at 70 kg/ha + lime (AN70L); and **10)** ammonium nitrate at 211 kg/ha + lime (AN211L).

### 2.3 Soil sampling and analysis

Soil samples were taken at the fall of 2020 where five subsamples were collected from 0-10 cm depth of each plot using an auger and mixed to make a composite sample. The samples were air-dried for five days, sieved (<2 ​mm) and stored in plastic jars for further analysis. Soil bulk density was determined using the core method with the samples collected from the middle of the plots using a steel core sampler with 7.5 cm diameter 5 cm height (Blake, 1965).

Soil samples were analyzed for exchangeable bases, total N, and pH using method described by Non-Affiliated Soil Analysis Committee (1990). Ammonium acetate (pH 7) was used for the extraction of exchangeable bases which were then quantified with the atomic adsorption spectrophotometry (Varian 2600). Soil pH was measured using a 1:2.5 ratio of soil: 1M KCL. Total N was measured using the LECO Trumac CNS Autoanalyser (2012).

Aggregate stability was determined using the wet sieving method as described by (Six et al., 2000a). Air-dried samples were sieved through an 8 ​mm sieve. An 80 g of 8 mm sieved soil sample was placed on a 2 mm sieve and submerged in a bowl with water for 5 min, followed by sieving for 2 min by moving the sieve up and down 50 times. The material remaining on the 2 mm sieve was transferred by backwashing into a pre-weighed pan and dried for 48 h at 60 °C. By repeating wet sieving using 250 μm, 53 μm sieves, four aggregate fractions were obtained. The water-stable soil aggregates (WSA) classes were separated into large macro-aggregates (LMA; > 2 mm), small macro-aggregates (MMA; > 250 μm–2 mm), micro-aggregates (MiA; 53–250 μm) and silt + clay fractions (SCA; < 53 μm). Mean weight diameter for WSA was used as a measure of aggregate stability. The MWD was calculated using the equation:

(1) $$MWD=\overset{n}{\underset{i=1}{\sum }}XiWi$$

(Abdalla et al., 2016) where *Xi* is the mean diameter for each fraction size, *Wi* is the proportional weight of the fraction from the total dry weight of soil used, and n is the number of aggregate classes separated. Organic C in water-stable soil aggregate fractions and bulk soil was analyzed using the Walkley- Black dichromate oxidation method (Walkley and Black, 1934). Carbon measured in each pool was then expressed as a percent of total SOC of bulk soil which allowed for the calculation of the percent recovery of soil C. The recovery of total SOC after fractionation was 87.7–99.5% across all treatments. Soil carbon stocks in bulk soil were calculated using the formula

(2) $${\text{SOC}}_{\text{stocks}}=\overset{n}{\underset{i=1}{\sum }}BDi.SOCconc.Depth_{volume}$$

(Don et al., 2007) where SOC_stocks_ = soil organic carbon stocks (t.ha^−1^), BD = Bulk density (g.cm^−3^), SOC_conc_ = soil organic carbon concentration (g/kg) and depth_volume_ is the sampling depth (cm).

### 2.4 Statistical analysis

Statistical analysis was done using GENSTAT 18^th^ edition. One-way analysis of variance (ANOVA) was used to compare effects of different treatments on studied soil properties. Treatment means were compared using Turkey's multiple comparisons with a significant difference at p < 0.05.

## 3 Results

### 3.1 Soil pH, organic carbon, nitrogen and aggregate stability

Long-term nitrogen addition and liming significantly (p < 0.05) altered pH, total N and MWD. The addition of N fertilization as ammonium sulphate significantly decreased soil pH irrespective of rate (Table 1). Only the higher application rate of ammonium nitrate (AN211) significantly decreased soil pH compared to the control. Lime (L) and all lime and nitrogen fertilizer combinations, except AS211L, significantly increased soil pH (p < 0.001) compared to the control (Table 1). Soil organic C content was not significantly altered by liming and N addition when compared with the control. Carbon stocks ranged from 43.4 to 50.6 tons C/ha across all treatments. Total soil N in AN211 (4.15 g N/kg) was comparable to AS70 and AS211 and all were significantly higher than AN70 and control (Table 1).

**Table 1:** Analysis of variance for different N and liming application on pH, exchangeable bases, organic carbon, total nitrogen and mean weight diameter.

Treatment	pH	Exch. Ca^2+^	Exch. Mg^2+^	Exch. K^+^	SOC	C stocks	Total N	MWD
		(cmol^+^/kg)			(g/kg)	(t/ha)	(g/kg)	(mm)
Control	4.31^b^	7.71^c^	4.00^bc^	0.56	49.7^ab^	43.34	3.11^a^	3.66^bc^
L	6.56^d^	17.04^d^	4.90^c^	1.09	50.4^ab^	46.8	3.39^abc^	4.03^c^
AS70	3.54^a^	2.27^ab^	0.83^a^	0.77	57.2^b^	50.6	3.98^cd^	2.01^a^
AS211	3.27^a^	1.03^a^	0.28^a^	0.74	56.1^b^	47.8	3.99^cd^	3.01^abc^
AN70	4.11^b^	6.36^bc^	3.32^b^	1.00	46.6^a^	44.5	3.57^ab^	2.67^ab^
AN211	3.61^a^	3.07^ab^	1.52^a^	0.73	57.6^b^	46.5	4.15^d^	2.27^a^
AS70L	6.05^c^	14.52^d^	4.82^c^	0.87	50.2^ab^	47.7	3.57^abcd^	3.51^bc^
AS211L	3.52^a^	5.38^bc^	1.31^a^	0.51	53.3^ab^	45.5	3.89^bcd^	2.84^ab^
AN70L	6.38 ^cd^	14.72^d^	4.58^bc^	0.85	49.1^ab^	44.9	3.42^abc^	3.61^bc^
AN211L	5.97^c^	15.30^d^	4.74^bc^	0.57	50.6^ab^	46.6	3.83^bcd^	3.58^bc^
**P value**	<**.001**	<**.001**	<**.001**	**0.556**	**0.003**	**0.976**	<**.001**	<**.001**

Values followed by a different letter in each column are significantly different (p < 0.05) according to Tukey's LSD procedure. C = control, L = lime (2250 kg ha^−1^), AS70 = ammonium sulphate at 70 kg ha^−1^; AS211 = ammonium sulphate at 211 kg ha^−1^; AN70 = ammonium nitrate at 70 kg ha^−1^; AN211 = ammonium nitrate at 211 kg ha^−1^; AS70L = ammonium sulphate at 70 kg ha^−1^ + lime; AS211L = ammonium sulphate at 211 kg ha^−1^ + lime; AN70L = ammonium nitrate at 70 kg ha^−1^ + lime and AN211L = ammonium nitrate at 211 kg ha^−1^ + lime. Exch.- exchangeable.

Mean weight diameter was only significantly decreased by the AS70 and AN211 treatments compared to the control (Table 1). While liming did not significantly affect MWD when compared to the control, the MWD in the L treatment (4.03) was significantly higher than in the AS70 (2.01), AN70 (2.67), AN211 (2.27), and AS211L (2.84).

Exchangeable Ca^2+^ and Mg^2+^ concentration were affected by lime and N addition (p < .001). The Ca^2+^ concentration ranged from 1.03 to 17.04 cmol^+^/kg across the treatments, while Mg^2+^ ranged from 0.28 to 4.90 cmol^+^/kg. Limed treatments significantly increased the concentration of exchangeable Ca^2+^ except AS211L compared to the control, while AS70 (2.27 cmol^+^/kg), AS211 (1.03 cmol^+^/kg) and AN211 (3.07 cmol^+^/kg) significantly decreased exchangeable Ca^2+^ when compared to the control (7.71 cmol^+^/kg) (Table 1). Exchangeable Mg^2+^ was lowered by AS70 (0.83 cmol^+^/kg), AS211 (0.28 cmol^+^/kg), AN211 (1.52 cmol^+^/kg) and AS211L (1.31 cmol^+^/kg) treatments (Table 1). Only the Lime (4.90 cmol^+^/kg) and AS70L (4.82 cmol^+^/kg) treatments had higher exchangeable Mg^2+^ concentration than the control. We found that neither long-term N addition nor liming affected exchangeable K^+^. Exchangeable bases followed the order Ca^2+^ > Mg^2+^ > K^+^ in all treatments.

### 3.2 Weight of water-stable aggregate fractions

**Figure 2:**
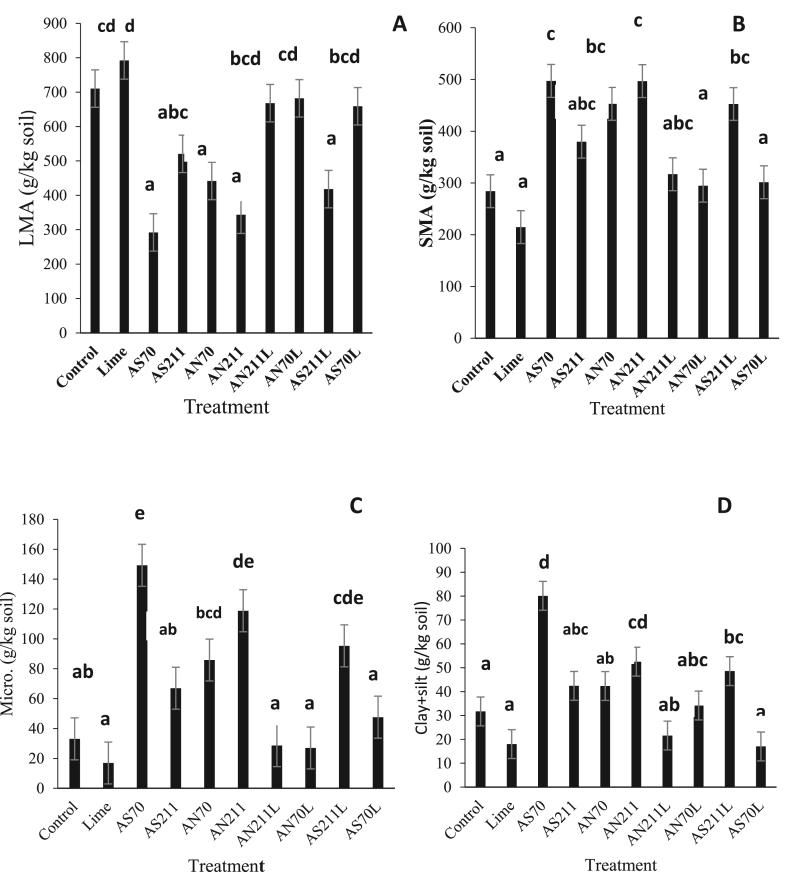
Masses of large macro-aggregates (A) (LMA; >2 mm), (B) Small macro-aggregates (C) micro-aggregates, and (D) silt + clay fractions for different treatments. Error bars represent standard error (n = 3).

Long-term nitrogen fertilizer and liming application affected water-stable aggregates (Figure 2). Except for AS211, all nitrogen fertilizer treatments had significantly lower large-macro-aggregates (LMA; >2 mm) fractions compared to the control (Figure 2A). Separate lime application did not affect water stable aggregates. Except for the AS211L, the combination of lime with nitrogen fertilizer treatments had higher large macro-aggregates when compared with the N fertilizer treatments without lime. The fertilizer treatments without lime, except the AS211, increased SMA (>250 μm–2 mm) when compared with the control (Figure 2B). The results of micro-aggregates (MiA; 53–250 μm) (Figure 1C) and clay + silt fractions (Figure 2D) followed the same trend as that of SMA (Figure 2B). Both AS70 and AN211 had higher MiA and clay + silt fractions compared to the control. Only MiA fraction was higher than the control in AN70 treatment. The addition of lime to the fertilizer treatments decreased the SMA and MiA fractions except in the AS211L wherein both fractions increased. Similarly, the combination of lime with AS70 and AN211 decreased the clay + silt fractions, while liming the AS11 treatment increased this fraction compared to the same treatments without lime. The WSA were in the order; (LMA; >2 mm) > (SMA; >250 μm–2 mm) > (SCA; < 53 μm) > (MiA; 53–250 μm), with the macroaggregate (>250 μm) fractions making up >50% of the total soil weight in all treatments.

### 3.3 Organic carbon in water-stable aggregates

The SOC content in the AS70 and AN211 treatments was significantly lower in the LMA fraction and higher in the SMA when compared to the control (Table 2). Lime and lime+ N fertilizer treatments did not significantly change carbon concentration in differently sized aggregates compared to the control (Table 2). However, lime and N fertilizer combination treatments had significantly higher C in all aggregate fractions compared to the same treatments without lime. Overall the control, L, AS70L, and AN70L, and AN211L had >3% C stored in the LMA fraction (Table 2). In all treatments, more than 60 % of SOC was stored in large macro-aggregates except for AS70, AN70, AN211, and AS211L where more carbon was stored in small macro-aggregates (>45 % of SOC). The results of SOC in the other fractions followed an inverse trend to that in the LMA, where the AS211L, AN211, and AS70 treatments significantly increased C in micro aggregate (53–250 μm) fraction in the order; AS211L < AN211 < AS70. Limed plot had lowest C content in MiA; 53–250 μm (0.09 %) compared to AS70 (2.67%), AN211 (2.49%) and AS211L (2.31%). Clay and silt had the least carbon content of all the aggregate fractions of which only AS70 had a significantly higher concentration compared to control (Table 2). Both micro aggregates and clay and silt fraction (<250 μm) stored less than 20% of the total C. There was a significant difference between L and nitrogen fertilized plots, where nitrogen fertilized plots increased C with an increase in N level in clay + silt compared to limed plots (Table 2).

**Table 2:** Organic carbon in water-stable aggregates.

Treatment	% Organic C in WSA			
	LMA; >2 mm	SMA; >250 μm–2 mm	MiA; 53–250 μm	SCA; < 53 μm
Control	3.40^cd^	1.26^ab^	0.17^abc^	0.13^abc^
Lime	3.65^d^	0.96^a^	0.09^a^	0.079^ab^
AS70	1.52^a^	2.67^c^	0.86^e^	0.37^d^
AS211	2.58^abcd^	2.02^abc^	0.47^cd^	0.24^cd^
AN70	1.91^abc^	2.02^abc^	0.39^bcd^	0.15^abc^
AN211	1.67^ab^	2.49^c^	0.65^de^	0.25^cd^
AS70L	3.41^cd^	1.35^ab^	0.22^abc^	0.064^a^
AS211L	2.08^abcd^	2.31^bc^	0.53^d^	0.22^bc^
AN70L	3.34^bcd^	1.58^abc^	0.15^ab^	0.15^abc^
AN211L	3.07^abcd^	1.48^abc^	0.12^ab^	0.067^ab^
**p value**	<**.001**	<**.001**	<**.001**	<**.001**

Values followed by a different lowercase letter in the same column are significantly different (p < 0.05) according to Tukey's LSD procedure. C = control (0 kg/ha), L = lime (2250 kg ha^−1^), AS70 = ammonium sulphate at 70 kg ha^−1^; AS211 = ammonium sulphate at 211 kg ha^−1^; AN70 = ammonium nitrate at 70 kg ha^−1^; AN211 = ammonium nitrate at 211 kg ha^−1^; AS70L = ammonium sulphate at 70 kg ha^−1^ + lime; AS211L = ammonium sulphate at 211 kg ha^−1^ + lime; AN70L = ammonium nitrate at 70 kg ha^−1^ + lime and AN211L = ammonium nitrate at 211 kg ha^−1^ + lime.

## 4 Discussion

Long-term nitrogen fertilizer addition resulted in significant soil acidification as pH ranged from 3.27-3.61 in N fertilized plots compared to 4.11 in the control. Similar results have been reported in several studies on the effect of long-term application of ammonium-based fertilizer (Malhi et al., 1998; Rasmussen and Rohde, 1989; Xu et al., 2020). Long-term application of nitrogen decreased soil pH in 0–10 cm depth by >16%. Ammonium based fertilizers are known to acidify the soil due to nitrification of NH_4_^+^ which releases H+ ions, further reducing the pH of this acidic soil (Are et al., 2018; Bouman et al., 1995). The greater acidifying effect of ammonium sulphate than of ammonium nitrate was due to the higher ammonium added in former than the latter at a given application rate. This has serious implications for grassland species composition and diversity which has been shown to decrease under nitrogen addition (Fang et al., 2012; Midolo et al., 2019; Ward et al., 2017c).

High total N concentration may be explained by significant amounts of N added (70 kg N/ha and 211 kg N/ha) annually via fertilization and reduced nitrification due to N-induced acidity. The recorded increase in soil N with increasing N rates for both N fertilizers is coherent with earlier findings (Ward et al., 2017a). Nitrification process is sensitive to acidic soils and limited by high Al and Mn or H^+^ concentrations (Robson and Abbott, 1989).

While some authors reported decline (Luo et al., 2019), increase (Eze et al., 2018; Fornara and Tilman, 2012; Xu et al., 2020) in SOC concentration and C stocks in grasslands with N addition and liming, SOC was not significantly affected by these treatments in this study. Current findings are consistent with earlier results from Ukulinga grassland experiment (Zeglin et al., 2007). Lack of SOC response may be attributed to possible carbon saturation. Studied soils have very high C, ranging from 49.7 g/kg to 57.6 g/kg across treatments, which may have decreased bulk soil C stabilization (Six et al., 2006). Carbon saturation prevents addition of new SOC even in fertilized plots with high C inputs. High soil carbon stocks in the surface layer of most grassland soils cause carbon saturation deficit to be relatively low therefore limiting the increase in soil carbon due to carbon inputs from residues (Whitehead et al., 2018). Angers et al. (2011) showed that soil pH and clay and silt particles are strongly related to carbon saturation deficit. Our soils were acidic and had a moderate proportion of clay and silt, which could also support carbon saturation (Angers et al., 2011). While Are et al. (2018) showed a positive correlation between SOC and aggregate stability, the relationship of SOC with aggregate stability was not significant in this study. However, aggregate size distribution and the SOC stored in the differently sized aggregate fractions were affected by N fertilizer application.

Nitrogen addition decreased the proportion of large macro-aggregates and increased micro-aggregates fractions which reduced mean weight diameter. This was due to the acidifying effect of N which led to rapid depletion of Ca^2+^ and Mg^2+^ (Table 1) cations playing an important role in aggregation through flocculation of clay particles (Rengasamy and Marchuk, 2011). Furthermore, ammonium-based fertilizers release NH_4_^+^ (with low hydration energy) to the soil which is adsorbed in place of high hydration energy cations (e.g. Ca^2+^ and Mg^2+^) causing dispersion of soil aggregates as the interlayer collapses (Rigol et al., 1999). N-induced acidity may have reduced nitrification making NH_4_^+^ a predominate form of N in studied plots. As such, NH_4_^+^-induced dispersion may also explain reduced fraction of large macroaggregates and MWD under separate N fertilization (Table 1). Similarly, Blanco-Canqui and Schlegel (2013) reported a decrease in aggregate stability upon addition of N at >100 kg N/ha/yr. Bai et al. (2020) found a strong correlation between MWD and exchangeable Mg^2+^ and fungal biomass. The acidity caused by increased N fertilizer application rate has been shown to reduce fungal biomass, root growth and length responsible for excreting binding agents.

Conversely, liming increased the soil pH towards neutral (pH 6.56). However, its effect was less significant when combined with the higher application rate of ammonium sulphate fertilizer due to the H^+^ released through nitrification. Liming also increased basic cations (Table 1) thus encouraging the formation of cation bridging between negatively charged clay surfaces and organic compounds (Bai et al., 2020). However, the effect of lime on WSA was only evident when applied in combination with N fertilizers. This suggests that an increase in soil pH when lime was led to improved conditions for nitrification thus decreasing NH_4_^+^ concentration. This may have counteracted the N-induced effects on soil aggregation, especially where lime was combined with AS70 and AN211. These results imply that liming helps maintain a stable soil structure while N addition can make the soil susceptible to erosion and degradation. Interestingly, AS211L did not increase MWD as expected with lime addition, instead, it reduced LMA, possibly due to a greater acidifying effect than the other N fertilizer treatments.

Several studies recorded higher SOC in macro-aggregates than in micro-aggregates after long-term application of mineral and/or organic fertilizers (Bhattacharyya et al., 2010; Tripathi et al., 2014). Generally, 80% of the total C is associated with macro-aggregates (>250 μm) in the upper soil layers of grasslands (Chen et al., 2019; Schwendenmann and Pendall, 2006). Although SOC in bulk soil was not affected by treatments, it can be said that SOC was redistributed into smaller fractions upon the breakdown of aggregates rather than being lost by decomposition suggesting that C dynamics were driven more by fertilizer and liming induced effect on aggregates. Applying N fertilizer without lime led to more carbon in SMA, MiA and SCA (Table 2) possibly due N-induced acidity and oxides rather than organic material being the primary binding agents. This high C in micro-aggregates suggests long-term storage of C as it is generally protected from microbial attack and thus have longer resident time compared to that stored in macro-aggregates (Six et al., 2000b).

Lime and control treatments had significantly higher mean values of OC in the LMA while their SMA and micro-aggregate fractions had lower OC compared to N fertilized plots. These results are consistent with the concept of aggregate hierarchy, which states that increasing aggregate class size results in an increase in C concentration in which micro-aggregates are bound together into macro-aggregates by binding agents (Six et al., 2000b; Tisdall and Oades, 1982). It seems that liming was effective as a control of N-induced acidity and NH_4_^+^ toxicity as no effects were observed when lime was applied separately.

## 5 Conclusion

The findings of this study showed that long-term N addition resulted in soil acidification and redistribution of organic C in water-stable aggregates at Ukulinga grassland. Nitrogen fertilizer, particularly ammonium sulphate, resulted in increased acidity and total soil N, reduction in both aggregate stability and exchangeable Ca and Mg. Liming had a reverse effect on soil pH and exchangeable bases but no effect on total soil N and MWD (except when applied in combination with N fertilizers). Total SOC did not respond to N fertilization and liming suggesting that studied soils may be carbon saturated thus limiting addition of new SOC from residue inputs. Separate application of N fertilizers decreased large macro-aggregates and increased micro-aggregates while separate liming had no effect on WSA. Despite lack of response of bulk soil C, N addition and liming resulted in redistribution of SOC in differently sized aggregate fractions. Nitrogen addition had a strong influence on labile organic carbon which was shifted to stable soil fractions confirmed by high C in SMA and micro-aggregates. Liming alone did not affect C associated with water stable aggregates while combination of lime with N fertilizers increased C associated with labile fractions and lowered it in stable fractions compared to separate N fertilization. This suggests that induced soil pH changes were the main driver of SOC redistribution and shift in WSA distribution due N fertilization and liming at Ukulinga grassland experiment. Carbon associated with differently sized aggregates may thus be used as diagnostic indicators of management induced changes in SOC. Liming may be used to ameliorate N-induced effects on soil pH and structure when N fertilizers are applied on grasslands. This will ensure that while the goal is mainly to improve grassland productivity using N fertilizers, caution is also taken to maintain good soil condition.

## Declarations

### Author contribution statement

Nkosinomusa Buthelezi-Dube: Conceived and designed the experiments; Contributed reagents, materials, analysis tools or data; Wrote the paper.

Kwenama Buthelezi: Performed the experiments; Analyzed and interpreted the data; Contributed reagents, materials, analysis tools or data; Wrote the paper.

### Funding statement

This work was supported by 10.13039/501100001321National Research Foundation (122198).

### Data availability statement

Data will be made available on request.

### Declaration of interests statement

The authors declare no conflict of interest.

### Additional information

No additional information is available for this paper.
